# Structural and functional characterization of genes *PYL-PP2C-SnRK2s* in the ABA signalling pathway of *Cucurbita pepo*

**DOI:** 10.1186/s12864-024-10158-9

**Published:** 2024-03-11

**Authors:** Jessica Iglesias-Moya, Álvaro Benítez, María Segura, Sonsoles Alonso, Dolores Garrido, Cecilia Martínez, Manuel Jamilena

**Affiliations:** 1 Department of Biology and Geology. Agri-food Campus of International Excellence (CeiA3) and Research Center CIAIMBITAL, University of Almería, 04120 Almería, Spain; 2 Department of Plant Physiology. Faculty of Science, University of Granada, 18021 Granada, Spain

**Keywords:** Zucchini squash, Response to ABA, Cold stress response, Germination, Tissular expression

## Abstract

**Background:**

The core regulation of the abscisic acid (ABA) signalling pathway comprises the multigenic families *PYL*, *PP2C,* and *SnRK2*. In this work, we conducted a genome-wide study of the components of these families in *Cucurbita pepo*.

**Results:**

The bioinformatic analysis of the *C. pepo* genome resulted in the identification of 19 *CpPYL*, 102 *CpPP2C* and 10 *CpSnRK2* genes. The investigation of gene structure and protein motifs allowed to define 4 PYL, 13 PP2C and 3 SnRK2 subfamilies. RNA-seq analysis was used to determine the expression of these gene families in different plant organs, as well as to detect their differential gene expression during germination, and in response to ABA and cold stress in leaves. The specific tissue expression of some gene members indicated the relevant role of some ABA signalling genes in plant development. Moreover, their differential expression under ABA treatment or cold stress revealed those ABA signalling genes that responded to ABA, and those that were up- or down-regulated in response to cold stress. A reduced number of genes responded to both treatments. Specific *PYL*-*PP2C*-*SnRK2* genes that had potential roles in germination were also detected, including those regulated early during the imbibition phase, those regulated later during the embryo extension and radicle emergence phase, and those induced or repressed during the whole germination process.

**Conclusions:**

The outcomes of this research open new research lines for agriculture and for assessing gene function in future studies.

**Supplementary Information:**

The online version contains supplementary material available at 10.1186/s12864-024-10158-9.

## Background

Plant growth and development, ranging from seed germination to fruit growth and maturation requires the orchestration of a multitude of factors, including phytohormones. Abscisic acid (ABA) plays an essential role in response to environmental stresses, regulating the relocation of resources to cope with stress, even at the expense of reducing plant growth [1]. Stress-induced ABA causes multiple changes at physiological and developmental levels, including stomatal closure, cuticular wax accumulation, leaf senescence, bud dormancy, growth inhibition, and the control of seed development, desiccation tolerance, dormancy and germination, among others [2]. In addition to its master role in stress response, ABA is a positive or negative regulator of developmental processes such as root growth and architecture [3], hypocotyl growth [4], and fruit growth, and maturation [5]. These functions are accomplished by itself or in interplay with other hormones. Therefore, understanding the genes involved in the ABA signalling pathway is essential not only to unravel the complex system that controls plant responses under stress, but also the role of this hormone in vegetative and reproductive development.

The ABA signalling pathway comprises three main components: the ABA receptors PYRABACTIN RESISTANCE 1/PYR1-LIKE/REGULATORY COMPONENTS OF ABA RECEPTORS (PYR1/PYL/RCARs) [6–8], the PROTEIN PHOSPHATASE TYPE-2C (PP2C) co-receptors [9–14], and SUCROSE NON-FERMENTING 1-RELATED SUBFAMILY 2 KINASES (SnRK2) [15–17]. In the absence of ABA, PP2Cs bind to and repress SnRK2s, thereby blocking the ABA signalling pathway [6–8]. In the presence of ABA, ABA binds to a PYR1/PYL/RCAR receptors (hereafter referred as PYLs), which undergoes conformational rearrangements that lead to the formation of PYL-PP2C heterodimer [6–8, 18, 19]. The interaction between PYL-ABA and PP2Cs provokes the dissociation of the PP2C-SnRK2, inhibiting the phosphatase activities of PP2Cs, which results in the autophosphorylation and the activation of SnRK2, and in the stimulation of ABA response [7, 8]. How the main components of the core ABA signalling system are regulated determines the response to ABA and is essential for maintaining plant growth under nonoptimal environments [2].

Several studies have identified genes that encode crucial components of ABA signalling in different species. The *PYL* family comprises 14 genes in Arabidopsis, 13 in rice, 24 in banana, 15 in tomato*,* and 14 in cucumber [20–23]. In Arabidopsis, *PYL* members have redundant functions in the regulation of PP2Cs, but differ in their ABA binding properties and their temporal and spatial expression [19, 24]. All of these are soluble proteins with STAR-RELATED LIPID-TRANSFER (START) domains that are distributed in the cytoplasm and nucleus [6, 7]. Although the phylogeny of PYLs leads to the establishment of three subfamilies [6], they can be classified into two major classes according to their oligomeric nature. Dimeric PYLs belong to subfamily III (PYR1 and PYL1–3, although PYL3 may have a faster equilibrium between dimer and monomer) [19], and monomeric PYLs belong to subfamilies I and II (PYL4–13, except for untested PYL7) [19, 25]. Dimeric ABA receptors have lower ABA binding affinity for dimer dissociation and inhibition of PP2C [19, 25], while monomeric forms have higher ABA binding affinity and can achieve complete inhibition of PP2C at much lower ABA concentration, or even in the absence of ABA [19]. Arabidopsis PYL13 is the only protein identified that does not respond to ABA and inhibits several PP2Cs in an ABA-independent manner [26, 27]. Genetic variability between PYL members provides explanation of their different basal activity and roles in plant development and stress responses [19, 25].

PP2Cs are Mg^2+^/Mn^2+^-dependent serine/threonine phosphatases that are closely related to the phosphoprotein phosphatases (PPP) family. They have, however, no sequence homology with PPP, and form a single cluster in the phosphoprotein metal phosphatases (PPM) family [28]. The PP2C family is highly conserved throughout evolution, having been found in archaea, bacteria, fungi, plants, and animals [29]. A total of 80 PP2Cs have been identified in Arabidopsis, 78 in rice, 87 in banana, 92 in tomato and 56 in cucumber [21, 28, 30, 31]. In Arabidopsis they have been divided into 13 subfamilies (A-L), with the exception of seven that could not be clustered [30]. Subfamily A contains proteins that have been characterized as key factors in the transduction of ABA signal, including ABI1, ABI2 (AT5G57050), AHG1 (AT5G51760), AHG3 (AT3G11410), HAB1 (AT1G72770), and HAB2 (AT1G17550) [32]. A-type PP2Cs inactivate SnRK2 by dephosphorylation, a function that is inhibited by PYL receptors in an ABA-dependent manner [30]. Subfamily B participates in the mitogen-activated protein kinase (MAPK) signalling pathway during salt stress or wounding [33]. Some genes in subfamily C, such as *AtPOL* (*AT2G46920*) and *AtPLL1* (*AT2G35350*), are involved in flower development and maintenance of stem cell polarity [34, 35]. Subfamily D members respond to salt and alkali stress [36] and are also involved in the regulation of seed germination in the dark, seed growth, and the ABA signalling pathway by mediating the activity of the plasma membrane H^+^-ATPase in cells [30, 37]. Subfamily E is involved in the regulation of stomatal closure [38]. Another member of PP2C of subfamily F, WIN2, (*AT4G31750*) is involved in the response of the plant to bacterial stress [39]. Only a few genes within each subfamily have been characterized, and information on the functions of PP2Cs from other subfamilies is not yet available.

The SnRK family comprises three major subfamilies, SnRK1, SnRK2, and SnRK3. SnRK1 is homologous to yeast SUCROSE NON-FERMENTING 1 (SNF1) kinase and mammalian AMP-ACTIVATED PROTEIN KINASES (AMPKs) [40], and is involved in cellular responses to nutritional signals [41], while SnRK2 and SnRK3 are specific to plants. SnRK3s are characterized by their ability to interact with Ca^2+^ sensor CALCINEURIN B-LIKE PROTEIN (CBL), while SnRK2 are the main drivers of ABA-triggered responses [42, 43]. SnRK2 kinases are monomeric serine/threonine protein kinases composed of a well-conserved N-terminal catalytic domain, and a regulatory C-terminal domain consisting of two subdomains: domain I and domain II [44]. Domain I, also known as the SnRK2 box, is conserved in all SnRK2s and is required for ABA-independent activation in response to osmotic stress. Domain II, also known as the ABA box, is required for ABA-dependent activation [44]. SnRK2 has been clustered into three subfamilies: subfamily I comprises kinases that are not activated by ABA; subfamily II comprises kinases that are not activated or are very weakly activated by ABA; and subfamily III comprises kinases strongly activated by ABA [45]. The SnRK2 family has been identified in many plant genomes, including 10 *SnRK2* genes in Arabidopsis [46], 11 in banana [21], 10 in rice [47], 11 in maize [48], and 11 in cucumber [49]. Gene expression data and mutant characterization in several species have shown that the *SnRK2* genes are an essential part of the ABA signalling pathway, at multiple stages of development and in response to abiotic stresses [50–52]. SnRK2s catalyze the phosphorylation of various downstream targets, including ABA INSENSITIVE 5 (ABI5), which plays an essential role in the post-germinative development arrest checkpoint [53–55], and ABA-RESPONSIVE ELEMENT BINDING FACTOR (ABF), which are transcription factors that finally induce the expression of ABA-responsive genes [7, 8, 14].

The squash, *Cucurbita pepo*, is an important vegetable crop with significant production and economic value around the world. Its genome was sequenced in 2018, which revealed a duplication of the whole genome associated with the origin of *Cucurbita* species [56]. We have identified 19 *PYLs*, 102 *PP2Cs* and 10 *SnRK2s* in the *C. pepo* genome, and determined their phylogenetic relationships, protein motifs, and gene structure. Their spatial gene expression patterns and transcriptional regulation during germination, as well as in response to ABA and cold treatments, were also investigated. This study will enhance our understanding of the core components of the ABA signalling pathway and offers a potential new perspective for squash breeding programs.

## Results

### Identification and clustering of *C. pepo* PYL-PP2C-SnRK2 proteins

A total of 19 *PYL*, 102 *PP2C*, and 10 *SnRK2* genes were identified by analyzing the *C. pepo* reference genome [56]. Tables S1-S3 include information on gene annotation for each *PYL*, *PP2C,* and *SnRK2* gene in the CuGenDBv2 and NCBI databases. Given that the current *C. pepo* genome derived from a complete genomic duplication, most of the identified ABA signalling genes had a paralogue on another chromosome. Only 4 out of 19 *CpPYLs*, 26 out of 102 *CpPP2Cs* and 2 out of 10 *CpSnRK2s* did not show the expected paralogous in the genome (Tables S1-S3).

**Fig. 1:**
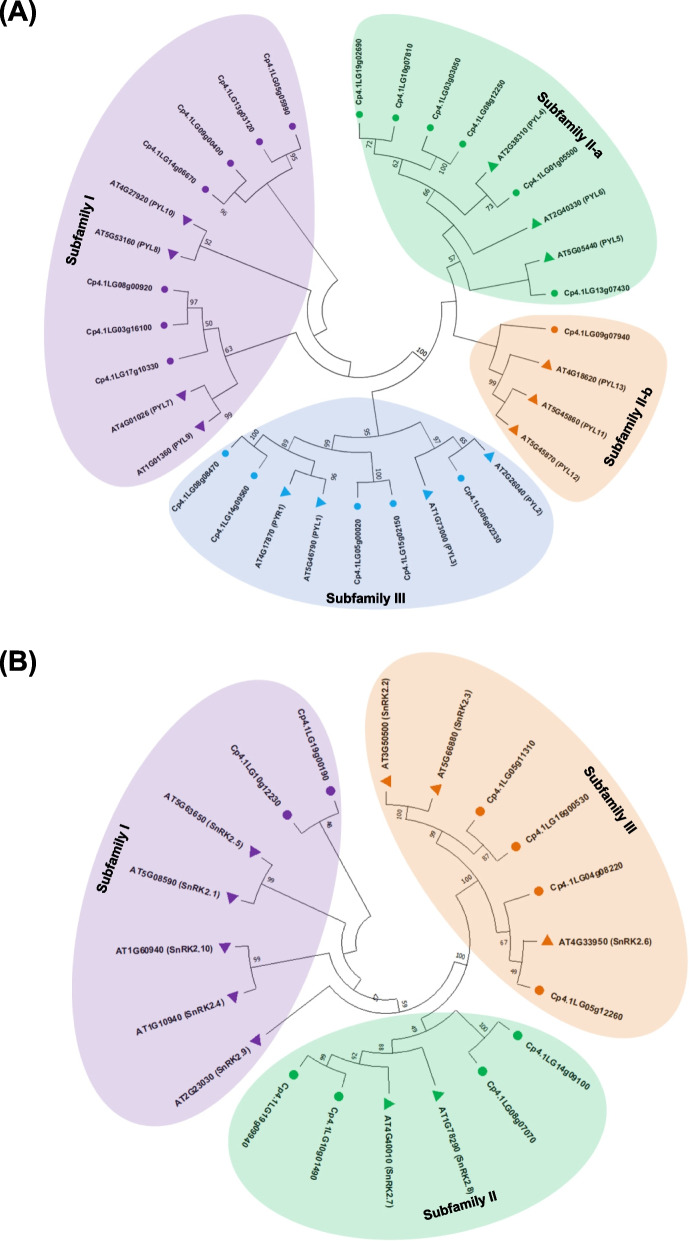
Phylogenetic analysis of *C. pepo* PYL (**A**) and SnRK2 (**B**) proteins. The circles represent squash proteins, and the triangles represent Arabidopsis proteins used for comparison. The phylogenetic tree was built with Mega X using the Maximum Likelihood method and 1000 bootstrap replications. The subfamily number is established according to the Arabidopsis classification adapted to this analysis [6, 45]. Members of Arabidopsis and squash included in the same clade were represented with the same color

**Fig. 2:**
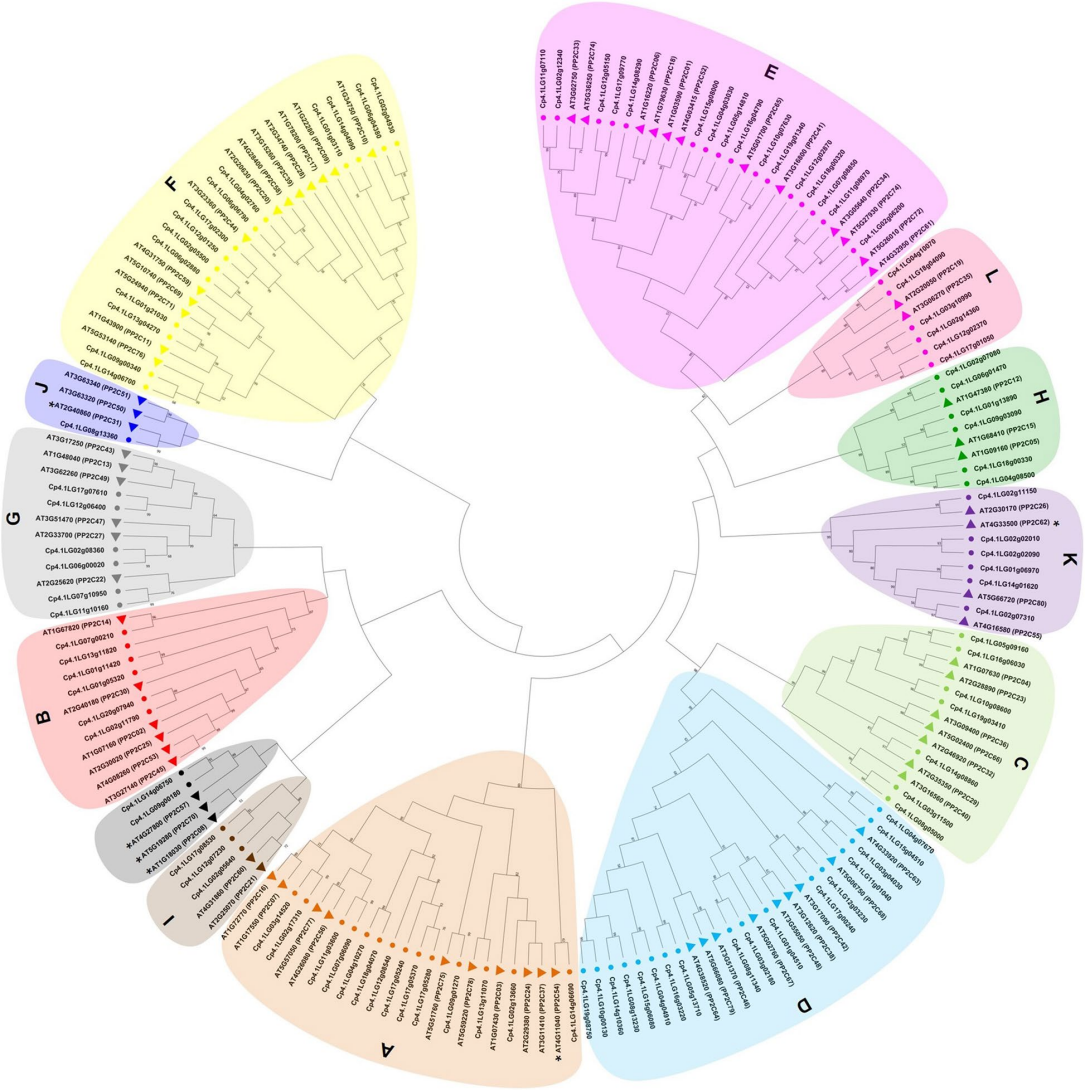
Phylogenetic analysis of *C. pepo* PP2C proteins. Circles represent squash CpPP2C proteins, and triangles represent Arabidopsis AtPP2C proteins used for comparison. Black asterisks indicate AtPP2C proteins that were unclustered in the phylogenetic analysis performed by Xue et al. [30]. The phylogenetic tree was built with Mega X using the Maximum Likelihood method and 1000 bootstrap replications. Subfamily letter is established in base to Arabidopsis classification adapted to this analysis [30]. Members of AtPP2C and CpPP2C included in the same clade were represented with the same color

To explore the evolutionary relationships and functional diversity of the CpPYL, CpPP2C, and CpSnRK2 proteins, we constructed phylogenetic trees using multiple sequence alignments of the PYL-PP2C-SnRK2 proteins from *C. pepo* and the model species Arabidopsis (Tables S1-S4). According to the phylogenetic analyses, the PYL, PP2C and SnRK2 families were divided into 4 (I-III), 13 (A-L, and one unclassified subfamily U), and 3 (I-III) subfamilies, respectively (Figs. 1 and 2). The CpPYLs were named on the basis of their homology with Arabidopsis PYL members [6]. The PYL subfamily I was found to be the largest, having 7 CpPYLs clustered together with AtPYL7–10, subfamily II-a included 6 CpPYL together with AtPYL4–6, and subfamily II-b only presented a single CpPYL protein (Cp4.1LG09g07940) that clustered with AtPYL11–13. Finally, subfamily III consisted of five CpPYL proteins clustered with AtPYR1 and AtPYL1–3 (Fig. 1A).

The phylogenetic tree of PP2C proteins of *C. pepo* and Arabidopsis showed the conserved diversity of this family of receptors (Fig. 2). From the 13 subfamilies found, subfamilies D and E are the largest ones, each one comprising 16 proteins, whereas subfamily J is the smallest, comprising only one protein. The AtPP2C proteins that were previously found unclustered [30], were here grouped in the subfamily A (AT4G11040), subfamily F (AT3G23360), subfamily J (AT2G40860), subfamily K (AT4G33500), and subfamily U (AT1G18030, AT4G27800, and AT5G19280), the later with the squash PP2C proteins Cp4.1LG14g06750 and Cp4.1LG09g00180 (Fig. 2). For the SnRK2 family, subfamilies II and III clustered four squash CpSnRK2 proteins each one, whereas only two squash proteins were found clustered in subfamily I, although this subfamily displayed the highest number of AtSnRK2s (Fig. 1B).

### Gene structure and conserved protein motifs of *C. pepo* PYL-PP2C-SnRK2s

**Fig. 3:**
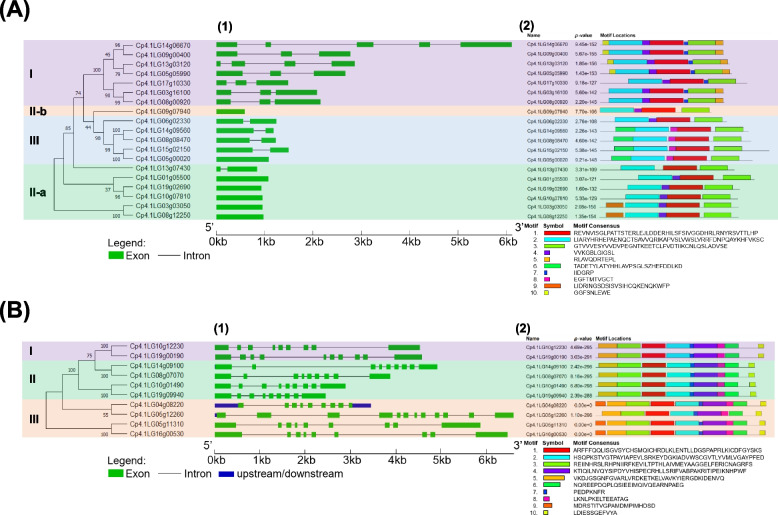
Gene structure and motif analysis of gene families *CpPYL* (**A**) and *CpSnRK2* (**B**). 1 Exon–intron structures of genes inferred with GSDS. The black lines and green boxes indicate introns and exons, respectively. The blue boxes indicate upstream and downstream untranslated regions (UTRs). 2 Distribution of protein motifs identified by MEME software. Each colored box represents a conserved motif sequence

**Fig. 4:**
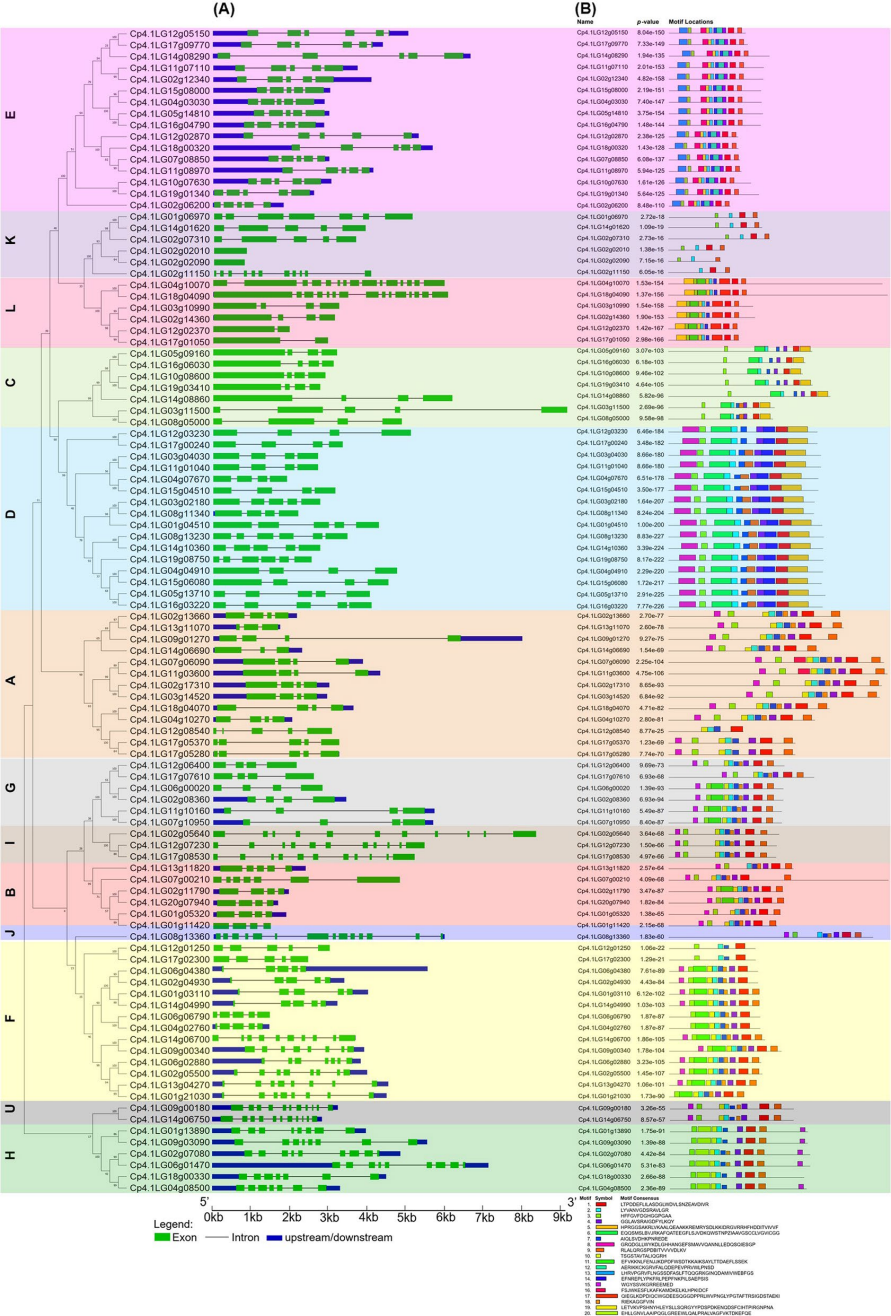
Gene structure and motif analysis of gene family *CpPP2C*. **A** Exon–intron structures of *CpPP2C* genes inferred with GSDS. The black lines and green boxes indicate introns and exons, respectively. The blue boxes indicate upstream and downstream untranslated regions (UTRs). **B** Distribution of all protein motifs identified by MEME software. Each colored box represents a conserved motif sequence

The gene structure of the *C. pepo PYL*-*PP2C*-*SnRK2* families was analyzed using the GSDS database (Figs. 3 and 4). The structure of the *CpPYL* gene subfamilies was in accordance with the phylogenetic analysis based on the sequence (Fig. 1A). Three to five exons were found within the genes of subfamily I. Members of subfamily II-a and II-b had one single exon, except for *Cp4.1LG13g07430* that presents two exons (Fig. 3A). The genes of subfamily III showed two exons with the sole exception of *Cp4.1LG05g00020*, which had only one (Fig. 3A). The conserved motifs in CpPYLs also supported their sequence homology (Fig. 3A). A total of 10 motifs were identified in members of the CpPYL family. All CpPYLs presented motifs 1, 2, and 3, while motif 7 was found to only be present in subfamily I (Fig. 3A). This is consistent with the annotation of the conserved domain PYR/PYL/RCAR_like (cd07821) in members of the CpPYL family (Table S1).

High diversity was found in the exon/intron structure of the *CpPP2C* genes (Fig. 4A). The number of exons within each subfamily was variable, although subfamilies E (5 exons), G (4 exons), I (11 exons), J (14 exons), and U (11 exons) displayed a fixed number of exons. All genes in the E, J, U, and H subfamilies, as well as some genes from subfamilies A, G, B, and F, presented annotated 3′ or 5′ untranslated regions (UTR) sequences, while the genes of the subfamilies K, L, C, and D did not have annotated 3′ or 5′ UTR sequences (Fig. 4B). The analysis of conserved motifs in this family led to the identification of as many as 20 (Fig. 4B). The presence/absence and the distribution of motifs within proteins are specific for each subfamily, validating the subfamilies established by phylogenetic analysis (Fig. 2). Motifs 1, 2, and 3 were significantly detected in almost all CpPP2C proteins except Cp4.1LG02g02090 and Cp4.1LG02g11150 of subfamily K and Cp4.1LG12g08540 of subfamily A. Most members of CpPP2C contained more than seven motifs, while some members had only four motifs, such as Cp4.1LG12g01250, Cp4.1LG17g02300, and members of subfamily K. Different subfamilies have their own specific motifs, probably in association with the functional divergence of each subfamily. So, all members of subfamily E comprised motifs 12 and 13, those belonging to subfamily L had specifically motifs 17 and 19, the subfamily D proteins contained motifs 8 and 14, and subfamily H was the only one containing motif 20 (Fig. 4B).

The gene structure of the *CpSnRK2* family is shown in Fig. 3B. Members of the three subfamilies had nine exons, except for *Cp4.1LG10g12230* and *Cp4.1LG05g11310*, which had 10 and 12 exons, respectively (Fig. 3B). As observed for *CpPYLs* and *CpPP2Cs*, *CpSnRK2* genes in the same subfamily also showed a similar exon-intron organization. The CpSnRK2 proteins comprised 10 motifs. All motifs but Nr. 9 were identified in all subfamilies, while motif 9 was only detected in three proteins in subfamily III (Fig. 3B). This is consistent with the conserved domains annotated in NCBI (Table S3). All members of the family, but three, had the *STKc_SnRK2* (cd14662) domain. The remaining three CpSnRK2, belong to subfamily III and presented *STKc_SnRK2–3* (cd14665) or *PKc_like* (cl21453) domains. STKc_SnRK2s are domains involved in plant response to abiotic stresses and ABA-dependent plant development, while *STKc_SnRK2–3* domains are representative of kinases strongly activated by ABA (Table S3).

### The expression profiles of *PYL-PP2C-SnRK2* genes in different plant organs

**Fig. 5:**
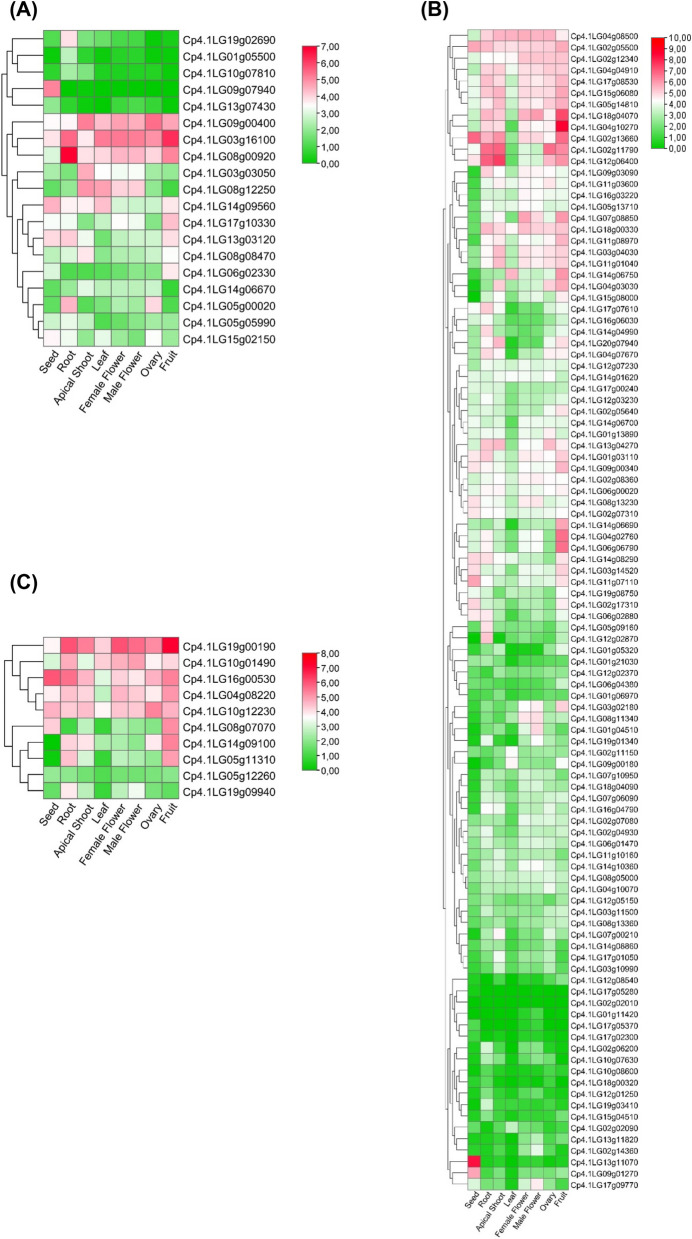
Expression profiles of *CpPYL-CpPP2C****-****CpSnRK2* genes in different plant organs. **A** Heatmap of *CpPYL* genes. **B** Heatmap of *CpPP2C* genes. **C** Heatmap of *CpSnRK2* genes. Data were normalized using log_2_ FPKM and TBtools was used to draw the expression heatmap. The expression values assigned to a color gradient from low log_2_ FPKM (green) to high log_2_ FPKM (red) are shown on the right of each figure

To gain insight into the role of each *PYL-PP2C-SnRK2* gene in the ABA signalling pathway, an RNA-seq analysis was performed in both vegetative and reproductive organs, including young roots and leaves, apical shoots, male and female flowers, and ovaries, fruits, and seeds. The number of raw reads and the percentage of reads after cleaning are listed in Table S5. Figure 5 shows the tissular expression patterns for each gene family.

#### CpPYLs

Most *CpPYLs* showed a low transcription level in the tissues under study. In general, the lowest FPKM values were found among *CpPYLs* in subfamily II-a (*Cp4.1LG19g02690*, *Cp4.1LG01g05500*, *Cp4.1LG10g07810*, *Cp4.1LG13g07430*) and the single gene of subfamily II-b (*Cp4.1LG09g07940*), which showed no or reduced expression in most tissues analyzed (Fig. 5A). Five *CpPYLs* of the subfamily III, and 4 *CpPYLs* of the subfamily I showed intermediate FPKM values (Log_2_ FPKM < 4.5), and the highest expression values were found for the genes *Cp4.1LG09g00400*, *Cp4.1LG03g16100*, and *Cp4.1LG08g00920* in subfamily I (Fig. 5A), which, together with their constitutive expression in the different organs, could indicate an essential role of these three *CpPYLs* in different physiological and developmental processes.

The up- and down-regulation of some *CpPYLs* in specific organs may be related to their functions. Thus, the specific up-regulation of Cp*4.1Lg09g07940* (Log2 FPKM > 5) in dry seed (Fig. 5A) suggests a potential association with the maintenance of desiccated and metabolically quiescent mature embryos. In the fruit, a high abundance of transcripts (Log_2_ FPKM > 4.5) of *Cp4.1LG03g16100*, *Cp4.1LG08g00920*, and *Cp4.1LG09g00400*, but also the specific activation of *Cp4.1LG17g10330* and *Cp4*.*1LG06g02330* was found (Fig. 5A), which may indicate the involvement of these genes in fruit growth and development. Similarly, roots were characterized by the highest transcript accumulation of C*p4.1LG08g00920* (Log_2_ FPKM > 6.8), and the specific activation of *Cp4.1LG19g02690* gene (Fig. 5A).

#### CpPP2Cs

Figure 5B shows the expression patterns of 102 *CpPP2Cs* in the eight analyzed plant organs. Most genes had values of Log_2_ FPKM < 4.5. In fact, 20 *CpPP2C* show Log_2_ FPKM < 3 in all tissues except in seed (Fig. 5B). Genes from different subfamilies of *PP2C* were clustered together based on gene expression, suggesting that their expression was not dependent on their phylogenetic origin. Female and male flowers presented similar transcription patterns for *CpPP2Cs*. Therefore, *Cp4.1LG07g08850, Cp4.1LG04g08500*, and *Cp4.1LG18g04070* were highly expressed (Log_2_ FPKM > 5) in both male and female flowers, and only a few genes, such as *Cp4.1LG01g04510* and *Cp4.1LG02g14360* were more expressed in male flowers than in female flowers (Fig. 5B)*.* Interestingly, the largest number of *CpPP2C* with a high expression level was found in the fruit, with a total of eight genes displaying Log_2_ FPKM > 6. Among them, *Cp4.1LG04g02760*, *Cp4.1LG06g06790*, and *Cp4.1LG02g1366* showed Log_2_ FPKM > 6.6; *Cp4.1LG18g04070* had Log_2_ FPKM > 7.3; and *Cp4.1LG04g10270* had a transcription value of Log_2_ FPKM > 8.5 (Fig. 5B). Regarding ovarian tissue, *Cp4.1LG02g11790* (subfamily B) had the highest abundance of transcripts (Log_2_ FPKM = 6.7), a gene that was also highly transcribed in fruits (Log_2_ FPKM = 6.2) (Fig. 5B).

In roots and apical shoots, the highest expression of the *PP2C* genes was found for the genes *Cp4.1LG12g06400* (subfamily G) and *Cp4.1LG02g11790* (subfamily B), showing Log_2_ FPKM > 6.7 (Fig. 5B). Finally, the leaf and seed showed a distinctive expression pattern with respect to other plant organs. Therefore, the genes *Cp4.1LG02g11790* (subfamily B) and *Cp4.1LG12g06400* (subfamily G) are specifically negatively regulated in leaves, while *Cp4.1LG04g08500* (subfamily H) and *Cp4.1LG014g06750* (subfamily U) (Log_2_ FPKM > 5) were specifically positively regulated in leaves (Fig. 5B). In dry seeds, the transcripts of *Cp4.1LG13g11070* (Log_2_ FPKM = 8.3), *Cp4.1LG02g13660* (Log_2_ FPKM > 6), and *Cp4.1LG09g01270* (Log_2_ FPKM = 5.7) were specifically and highly accumulated (Fig. 5B), suggesting that they were specifically expressed during seed maturation.

#### CpSnRK2s

The *CpSnRK2* genes were clustered in two groups according to their tissue transcription profiles (Fig. 5C), although they were not related to the phylogenetic subfamilies established by sequence homology. One of the clusters comprised genes with Log_2_ FPKM < 3.9, including *Cp4.1LG08g07070*, *Cp4.1LG14g09100*, *Cp4.1LG05g11310*, *Cp4.1LG05g12260* and *Cp4.1LG19g09940*. The other group showed values of Log_2_ FPKM between 2.5 and 7.2 and includes *Cp4.1LG19g00190*, *Cp4.1LG10g01490*, *Cp4.1LG16g00530*, *Cp4.1LG04g08220* and *Cp4.1LG10g12230*. Among the *CpSnKR2* genes*,* the gene *Cp4.1LG19g00190* showed the highest levels of expression in the different plant organs studied (Fig. 5C).

A low level of transcription was found for most of *CpSnRK2* in leaf, even for the most transcribed genes, *Cp4.1LG10g01490* (subfamily II) and *Cp4.1LG19g00190* (subfamily I), which showed transcription values of Log_2_ FPKM < 4.2 (Fig. 5C). In dry seeds, three *CpSnRK2* genes presented Log_2_ FPKM values > 4, with gene *Cp4.1LG16g00530* (subfamily III) showing the highest expression level (Log_2_ FPKM = 5.9) (Fig. 5C). In contrast, the genes *Cp4.1LG14g09100* (subfamily II) and *Cp4.1LG05g11310* (subfamily III) had the lowest abundance of transcripts in seed (Fig. 5C).

In fruit, the gene *Cp4.1LG19g00190* (subfamily I) showed the highest abundance of transcripts (Log_2_ FPKM = 7.2), and the other five were transcribed with Log_2_ FPKM > 4.9 (*Cp4.1LG16g00530*, *Cp4.1LG05g11310*, *Cp4.1LG08g07070*, *Cp4.1LG14g09100*, and *Cp4.1LG04g08220*) (Fig. 5C). In the ovary, only *Cp4.1LG16g00530*, *Cp4.1LG10g12230* and *Cp4.1LG19g00190* showed moderately transcribed values of Log_2_ FPKM > 4.6). In roots, many genes showed Log_2_ FPKM > 4 (*Cp4.1LG10g01490*, *Cp4.1LG04g08220*, *Cp4.1LG10g12230*, *Cp4.1LG14g09100* and *Cp4.1LG05g11310*), although the highest transcription values were found for *Cp4.1LG19g00190* (subfamily I) (Log_2_ FPKM = 6), followed by *Cp4.1LG16g00530* (subfamily III) (Log_2_ FPKM = 5.6) (Fig. 5C). The female and male flowers showed similar expression patterns. The highest transcription values were detected for *Cp4.1LG19g00190* (subfamily I), with Log_2_ FPKM values of 6.0 and 5.6 in female and male flowers, respectively. Regarding the apical shoots, only the genes *Cp4.1LG16g00530*, *Cp4.1LG04g08220*, *Cp4.1LG10g12230* and *Cp4.1LG19g00190* (subfamily I) had expression values of Log_2_ FPKM > 4 (Fig. 5C).

### The expression of squash *PYL-PP2C-SnRK2* genes in response to exogenous ABA and cold stress

Differential expression analysis was performed to study the putative functions of the core ABA signalling components in the leaves of *C. pepo* in response to ABA and cold. The number of raw reads and the percentage of reads after cleaning are shown in Table S6. The generated gene count matrix was used for Multidimensional Scaling (MDS) of the expression data of the 9 leaf samples, three replicates for either control, ABA, and cold treatments (Fig. S1). The MDS plot showed the overall clustering of samples based on gene expression patterns. Dimensions 1 and 2 explained 65 and 11% of the variation in gene expression, respectively. The samples were completely separated according to treatment and the three replicates of each sample were tightly grouped together, indicating that the experimental data are reliable for further analysis (Fig. S1).

To determine the transcriptomic changes in the *PYL*-*PP2C*-*SnRK2* genes that responded to cold stress and exogenous application of ABA, two pairwise comparisons were performed: ABA treatment versus control, and cold treatment versus control. Only genes with an adjusted *P*-value < 0.05 were considered DEGs (Table 1). 9 out of 19 *CpPYLs*, 46 out of 102 *CpPP2Cs*, and 3 out of 10 *CpSnRKs2* changed their expression in response to ABA and/or cold treatment. Most DEGs responded specifically to ABA or cold, but only a few responded to both treatments (Table 1).

**Table 1:**
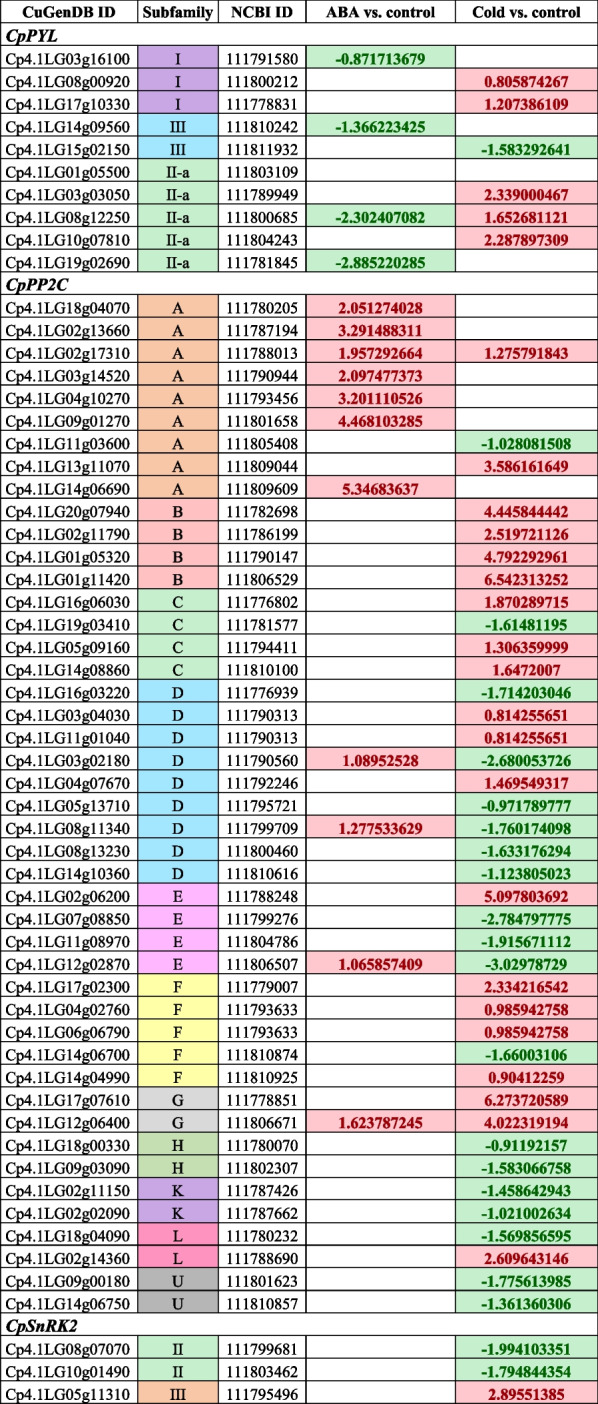
Differentially expressed ABA signaling genes in response to exogenous application of ABA and cold stress. Values are log_2_ (Fold Change) with an adjusted *p*-values < 0.05. Green and red shaded cells indicate negative and positive values for log_2_ (FC) in two pairwise comparisons: ABA vs. control and cold vs. control. Only log_2_ (FC) with an adjusted *P*-value < 0.05 are given

Treatment with ABA in *C. pepo* leaves led to the identification of 15 significant DEGs in the ABA signalling pathway, 4 down-regulated *CpPYLs* and 11 up-regulated *CpPP2Cs* (Table 1). No *CpSRK2* changed its expression in response to ABA treatment (Table 1). Three of the down-regulated *CpPYLs* belonged to subfamilies I and II, whose members encode monomeric receptors with high ABA-binding affinity in Arabidopsis, while only one of the down-regulated *CpPYLs* was of subfamily III, a dimeric receptor with lower ABA-binding affinity in Arabidopsis (Table 1). The genes most up-regulated of *CpPP2C* belonged to subfamily A (Table 1, Fig. 2), a group of *PP2Cs* that in Arabidopsis play a key role in ABA signal transduction, including *ABI1, ABI2* (AT5G57050), *AHG1* (AT5G51760), *AHG3* (AT3G11410), *HAB1* (AT1G72770), and *HAB2* (AT1G17550). These results indicate that only a few of the *C. pepo* genes in the ABA signalling pathway appear to be regulated by ABA. Furthermore, ABA regulation was very precise, specifically activating the transcription of certain *CpPP2Cs,* but inhibiting the transcription of certain *CpPYLs*.

Cold was also found to be involved in the regulation of ABA signalling genes. The cold treatment on leaves led to 48 significant DEGs in the ABA signalling pathway of *C. pepo*, 26 of which were up-regulated (5 *CpPYLs*, 20 *CpPP2Cs* and 1 *CpSnRK2*) and 22 were down-regulated (1 *CpPYLs*, 19 *CpPP2Cs*, and 2*CpSnRK2s*) (Table 1). In contrast to what was observed for ABA treatment, all *CpPYLs*, except for *Cp4.1LG15g02150*, were up-regulated in response to cold stress, but were not the same *CpPYLs* that those that were down-regulated by ABA (Table 1). Only the *CpPYL* gene *Cp4.1LG08g12250* was found to be significantly induced by cold and repressed by ABA in the treated leaves (Table 1). The up-regulated *PYLs* belonged to subfamilies I and II, whose members have a high ABA binding affinity in Arabidopsis, while the down-regulated one belonged to subfamily III, which in Arabidopsis is a receptor with a lower ABA binding affinity (Table 1). Cold treatment did not regulate *CpPP2C* genes in the same way, so some were induced by cold, and others were repressed by cold (Table 1). Most of the B members of the subfamily of *CpPP2C* were up-regulated by cold stress and showed high Fold Change (FC) values (Table 1). Other genes of *CpPP2Cs,* including *Cp4.1LG02g06200* from subfamily E and *Cp4.1LG17g07610* and *Cp4.1LG12g06400* from subfamily G, were also found to be highly up-regulated in response to cold stress (Table 1). Regarding the *CpSnRK2* genes, we found that one gene of subfamily III (*Cp4.1LG05g11310*) was significantly up-regulated in response to cold stress, while two genes from subfamily II (*Cp4.1LG08g07070* and *Cp4.1LG10g01490*) were down-regulated (Table 1).

In conclusion, the cold and ABA treatments in *C. pepo* leaves have shown a very different effect on the transcription of ABA signalling genes. Most DEGs were specific to one treatment or another, indicating that cold can regulate some of the genes in the ABA transduction pathway in an ABA-independent manner. Only 1 *CpPYL* and 5 *CpPP2C* genes changed their expression in response to both treatments (Table 1). However, three *CpPP2C* genes, *Cp4.1LG03g02180*, *Cp4.1LG08g11340* and *Cp4.1LG12g02870*, were up-regulated in response to ABA, but down-regulated in response to cold stress. The contrary is observed for a *PYL* gene, *Cp4.1LG08g12250* that was up-regulated under cold stress but down-regulated under ABA treatment (Table 1). Only two *CpPP2C* genes, *Cp4.1LG02g17310* and *Cp4.1LG12g06400*, were up-regulated in both cold and ABA (Table 1).

### Expression profiles of the *PYL-PP2C-SnRK2* genes during germination

A second differential expression analysis was performed during different stages and conditions of germination. Seed samples were taken in three stages: mature dry seed, seed soaked in water or ABA for 16 h, and seed at the emergence of the radicle under water or ABA treatment, which is considered the completion of germination. The number of raw reads, percentage of reads, percentage of quality reads, and mapped reads are listed in Table S7. Multidimensional scaling (MDS) plot of the expression data of the 15 samples is shown in Fig. S2. Biological replicates were found to be tightly grouped, but separated from other samples, which ensured data reliability for downstream analysis.

Differential gene expression was assessed through pairwise comparison between imbibed and dry seed, as well as between germinated and dry seed. This approach enabled the evaluation of transcriptomic changes for the *PYL*-*PP2C*-*SnRK2* genes following water or ABA soaking for 16 hours (imbibition phase) and throughout the entire germination process, which is defined by embryonic root emergence.

In response to seed imbibition in water, 28 ABA signalling genes were up-regulated, comprising 11 *CpPYLs*, 14 *CpPP2Cs*, and 3 *CpSnRK2s* and 21 were down-regulated, including 1 *CpPYL,* 19 *CpPP2Cs*, and 1 *CpSnRK2s* (Table 2). Imbibition of seeds in ABA resulted in similar FC, although the effects were reduced compared to imbibition in water (Table 2). Hence, the alterations in gene expression were primarily attributed to the reduction of endogenous ABA levels during seed imbibition, rather than the influence of applied ABA. According to this conclusion, a total of 26 Differentially Expressed Genes (DEGs) were identified in seed that was imbibed in either water or ABA, with 12 of them being specific to the water imbibition treatment (Table 2). Only 3 DEGs were specific to the ABA imbibition treatment (Table 2).

**Table 2:**
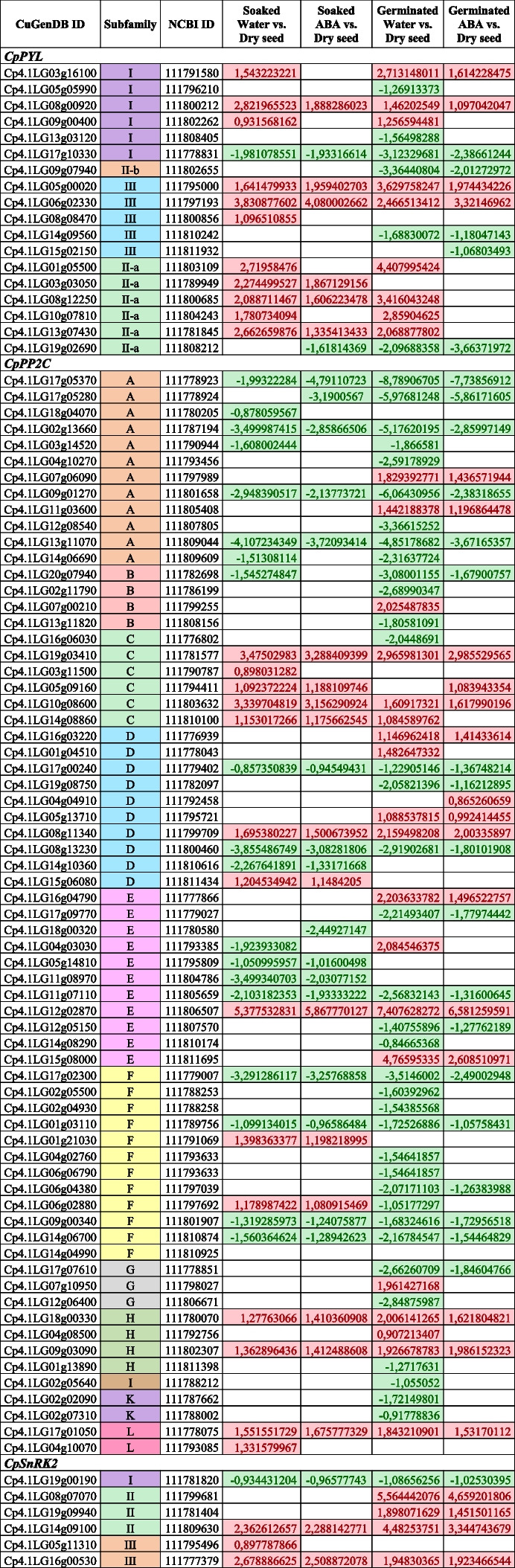
Differentially expressed ABA signaling genes at two stages of seed germination and under different treatments. Values are log_2_ (Fold Change) with an adjusted *p*-values < 0.05. Green and red shaded cells indicate negative and positive values for log_2_ (FC) in four pairwise comparisons: seeds soaked in water vs. dry seed, seeds soaked in ABA vs. dry seed, seeds germinated in water vs. dry seed, and seed germinated under ABA treatment vs. dry seed. Soaked seed corresponds to seed imbibed in water or ABA for 16 h, and seed was considered germinated at radicle protrusion. Only values of FC with an adjusted *p*-value < 0.05 are shown

The number of DEGS upon the completion of the germination and the onset of radicle protrusion was higher than that displayed after imbibition. During the later phase, 32 ABA signaling genes were up-regulated (9 *CpPYLs*, 18 *CpPP2Cs*, and 4 *CpSnRK2s*) and 43 were down-regulated (6 *CpPYLs*, 36 *CpPP2Cs*, and 1 *CpSnRK2s*) in the seeds germinated in water (Table 2). 44 DEGs were similarly regulated in seeds germinated in water or ABA, while 30 DEGs were specific for seeds germinated in water (Table 2). These data suggest that the effect of internal ABA, that decrease during germination, predominates over the effect of the external ABA treatment. Only 3 genes changed their expression when the seed germinated in presence of ABA but not in water (Table 2).

### The validation of gene expression results

**Fig. 6:**
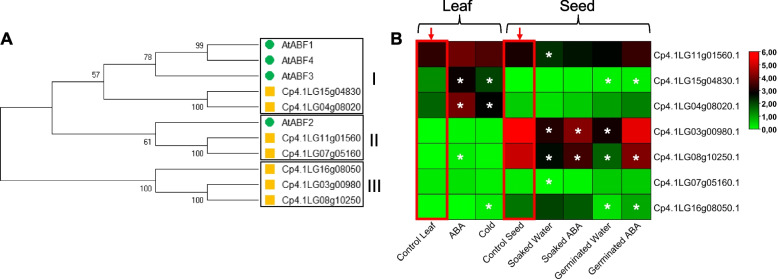
Expression of *ABF* genes. **A** Phylogenetic analysis of the *C. pepo* and Arabidopsis ABF proteins. The yellow squares represent squash proteins, and the green circles represent Arabidopsis proteins used for comparison. The phylogenetic tree was built with Mega X using the Maximum Likelihood method and 1000 bootstrap replications. **B** Expression profiles of *ABF* genes in leaf and seed under different treatments and stages of germination. Data were normalized using log_2_ FPKM and TBtools was used to draw the expression heatmap. The expression values assigned to a color gradient from low log_2_ FPKM (green) to high log_2_ FPKM (red) are shown on the right of figure. White asterisks indicate genes with significant differences in gene expression compared to a reference sample (red arrow) used as control (adjusted *p*-values < 0.05). For the leaf experiment, the reference control sample was the leaf of the untreated seedling growing under control conditions. For the seed experiment, the reference sample was the dry seed

To validate the effectiveness of treatments, we analyzed the expression of ABRE binding factors (ABF) genes that were reported to be regulated by ABA and cold in vegetative organs of Arabidopsis [57]. In squash, we identified 7 *ABF* genes (Table S8). The phylogenetic tree with the ABF proteins from squash and Arabidopsis showed three distinct branches (Fig. 6A). The first branch clustered two ABFs of squash and Arabidopsis. The second branch grouped AtABF2 and two squash ABFs. The third branch consisted of only squash ABFs.

RNAseq data confirmed that ABA and cold treatments performed in squash leaves were able to up-regulate the *Cp4.1Lg15g04830* and *Cp4.1LG04g08020* genes (Fig. 6A and Table S9). The gene Cp4.1LG08g10250 was only up-regulated in response to ABA treatment, while *Cp4.1LG16g08050* was only up-regulated in response to cold stress (Fig. 6A and Table S9). The seed results were very similar. Most of the *CpABF* genes were down-regulated after imbibition and germination in water (Fig. 6A and Table S9), indicating that these genes are positively regulated by ABA. As expected, the seed expression of *ABF* genes was not reduced to the same extent when imbibition and germination were carried out in the presence of ABA (Fig. 6B, Table S9).

It is worth mentioning that the expression level of *CpABF* genes depended on the tissue analyzed (Fig. 6). In leaves, the highest abundance of transcripts was found for *Cp4.1LG11g01560*, *Cp4.1Lg15g04830*, and *Cp4.1LG04g08020*, while in seeds the most abundant transcripts were those of *Cp4.1LG03g00980* and *Cp4.1LG08g10250*. These results strongly suggest that each squash *CpABF* gene has a role in germination or abiotic stress responses in the leaf.

## Discussion

### Evolutionary relationships between members of the PYL-PP2C-SnRK2 families in *C. pepo* and plant species

*PYLs*, *PP2Cs* and *SnRK2s* are encoded by multigene families with a variable number of genes among species. The number of *CpPYL* and *CpSnRK2* genes in *C. pepo* (19 and 10, respectively) is similar to that of Arabidopsis (14 and 10) or cucumber (14 and 11) [23, 46, 49]. However, the number of *CpPP2C* genes was higher in *C. pepo* (102) than in Arabidopsis (80) or cucumber (56) [28, 58], which is probably associated with duplication of the whole genome in *Cucurbita* [56, 59], but also with the evolutionary diversification of the *PP2C* gene family to adapt plant species to multiple environments [28, 60].

The classification of the three gene families agrees with previous phylogenetic analyses in Arabidopsis, *Brassica rapa*, rice, tomato, banana, or cucumber [20–23, 28, 30, 31, 46, 47, 49, 61]. Gene structure and protein motifs were also found to be conserved within members of the same family in *C. sativus*, rice and *Gossypium* ssp. [22, 23, 62], indicating a highly conserved function. Significantly, all 19 CpPYL proteins contained motif 1, 2 and - 3 (Fig. 3A), which is also present among CsPYLs, AtPYLs and OsPYLs [22, 23]. Other motifs were found to be specific to a species or a subfamily. Motif 7, was specific to the CpPYL subfamily I (Fig. 3A), suggesting an exceptional biological function linked to subfamily I.

The *PP2C* family is present in bacteria, fungi, plant, and animals, regulating stress signalling pathways. In plants, the diversity of structures found among PP2C in different species gave rise to a different function in signalling mechanisms [13, 29]. In *C. pepo*, this diversity was associated with changes in gene structure and protein motifs, similar to what was found in *C. sativus* [28] and other species.

The CpSnKR2 family was found to be less diverse than the CpPYL and CpPP2C families. Most of the *C. pepo* genes showed 9 exons, which is in agreement with the structure of the *SnKR2* gene in other species of dicot and monocot [63–65]. Furthermore, all motifs, except motif 9, were conserved in the three subfamilies. Common motifs are likely to preserve the more relevant functions of these proteins, including the N-terminal motif 1 with the active site signature of serine/threonine protein kinases, the N-terminal motif 5, which is an ATP binding signature [63, 64], and the C-terminal domains I (SnRK2 box) (motif 6) and II (ABA box) (motifs 8 and 10), which are required to respond to osmotic stress and ABA, respectively [66]. The presence of these domains and motifs indicates that all squash CpSnRK2 identified in this work are functional and ABA dependent.

### Specific spatial expression associated with the role of ABA signalling genes in the development of *C. pepo*

The expression profiles of *PYL-PP2C-SnRK2* gene families in different plant organs could clarify the divergent roles of the ABA signal transduction components during plant development and plant stress responses. The constitutive low accumulation of *CpPYLs, CpPP2C* and *CpSnRK2* transcripts in the tissues analyzed suggests that many of them are not involved in specific developmental processes. However, the seed-specific *CpPYL* gene *Cp4.1LG09g07940* of subfamily II-b was highly negatively regulated at germination (Table 2), similar to the pattern observed with *AtPYL11*, *AtPYL12* and *AtPYL13*, which are also specifically expressed in mature Arabidopsis seed and play important roles in ABA-mediated seed germination [67]. The highest transcription of *CpPYL* was observed for subfamily I genes, represented in Arabidopsis by the *AtPYL7* and *AtPYL9* genes*,* and *AtPYL8* and *AtPYL10*, which play a relevant role in reproductive and root development, respectively [22, 68–72].

The *Cp4.1LG13g11070* (subfamily A) was highly and specifically expressed in dry seed. This gene clustered with Arabidopsis *AT1G07430* (Fig. 2), which is a seed-specific PP2C that acts as one of the major negative regulators of seed dormancy [73]. Other PP2C were found to be strongly or specifically expressed in fruit and may play a role in fruit growth and development [21].

The highest transcription of the *CpSnRK2* genes was observed in fruit and dry seeds. *Cp4.1LG14g00190* belongs to subfamily I, which are important regulators of fruit ripening in Arabidopsis [74]. The high transcription of the *CpSNRK2* gene *Cp4.1LG16g00530* in seed, and its upregulation after imbibition and germination (Table 2), also corresponds to the described role of class II SnRK2 in Arabidopsis germination, dormancy, and seedling growth [45, 75].

### Abiotic stress response of ABA signalling genes in the vegetative organs of *C. pepo*

Some conclusions were drawn from leaf exposure to ABA and cold. Genes within the same family responded differently to ABA and cold, which is consistent with the specific regulation of ABA genes under different stresses in other plants [21, 61, 62, 64].

In response to ABA, *CpPP2C* were up-regulated while *CpPYLs* were down-regulated. The response of *PYL* genes to ABA is very variable [23, 62]. The subfamily-A *PP2C*s, which are known to be negatively regulated by ABA in different species [28–30], were those showing the highest expression changes in our experiment. Three of them were homologous to recognized negative regulators of ABA response in Arabidopsis: *ABI1* (*ABA insensitive 1*), *AHG3/PP2CA* (*ABA hypersensitive germination 1*), *HAB1* (*hypersensitive to ABA1*) [12, 76] and may play important roles in ABA-mediated processes.

In response to cold, specific *CpPYLs* and *CpSnRK2s* genes were up and down-regulated. This agrees with the variable regulation of *PYL* and *SnRK2* observed in other species to face cold stress [21, 22, 63, 77]. A higher number of *CpPP2C* genes were induced or repressed in response to cold. Unlike what was indicated for other species such as Arabidopsis, rice, or *Brachypodium distachyon* or banana [21, 29, 30], the subfamily A of *C. pepo* appears to be less important under cold stress. In contrast, 4 out of 6 genes of subfamily B and members of subfamily E and G were up-regulated in response to cold stress in squash. PP2Cs from subfamily B are activated by hyperosmolarity, salt, cold, or drought [78], while members of subfamily E are expressed in guard cells [79]. Little is known about the role of other PP2C subfamilies. However, squash data suggest a role for PP2C of subfamilies K and U in response to cold stress.

### Relevant ABA signalling genes in squash germination

Our results clearly demonstrated that genes of the ABA signalling pathway play a relevant role in germination [76, 80]. The number of DEG in the germinated seed was higher than in the imbibed seed, indicating that the mechanism driving radicle protrusion is partly established during the imbibition phase of germination [81]. Furthermore, the similarities in gene expression between seed soaked and germinated in water compared to seed soaked and germinated in ABA demonstrate that these changes are primarily controlled by the internal ABA content of seeds, which is progressively decreased during germination. In fact, external ABA treatment was only able to partially counteract some of the transcriptional changes in the ABA signaling genes.

The specific down-regulation of *Cp4.1LG09g07940*, the only member of *CpPYL* in subfamily II-b, suggests an important role for this gene in germination. *PYLs* from subfamilies I and II in orchids [82], and other *PYLs* from Arabidopsis (*AtPYR1*, *AtPYL1*–*5*, *AtPYL8*) also participate in germination [83]. Regarding *CpPP2C,* 11/13 DEGs in subfamily A and 11/14 DEGs in subfamily F were found to be mainly repressed during germination. Some members of subfamily A also showed a high down-regulation during germination. These results agree with the germination ability of mutants in the *PP2C* genes of subfamily A, including *abi1* and *abi2*, which display ABA insensitivity and reduced seed dormancy [9, 76]. The regulation found for the *CpPP2C* genes of subfamily F during germination suggests a role in the control of germination that has not been previously reported for this family in other species. Additionally, the induction of subfamily C *PP2C* genes, which play a significant role in cell polarity in Arabidopsis [34], also appears to also control germination in squash. Finally, five DEGs of the *CpSnRK2* family were down-regulated (subfamily I) and up-regulated (subfamily II and III) in the germinated seed. This is consistent with the positive role of SnRK2 in the ABA response. In Arabidopsis, triple mutants (*snrk2.2 snrk2.3 snrk2.6*) and double (*snrk2.2 snrk2.3*) mutants present growth defects during seed development, loss of dormancy, and elevated ABA content in seed, indicating that subfamily III genes are also required for proper seed germination [75, 84].

## Conclusions

A combination of genomic and transcriptomic analyses allowed for the identification and structural and functional characterization of a total of 19 *CpPYL,* 102 *CpPP2C* and 10 *CpSnRK2* genes. Analyzing the protein sequences, gene structures, and protein domains and motifs, was essential to differentiate the three multigenic families into different subfamilies, as defined in Arabidopsis and other model species. The RNAseq data indicate that the function of some subfamilies and genes was similar to that previously reported in Arabidopsis, but specific genes have been identified that play essential roles in the development of some organs, the germination process, or the plant’s response to ABA and cold stress. The results prove to be a valuable tool for functional genomics in crop species.

## Methods

### Identification of the *PYL-PP2C-SnRK2* genes in *C. pepo* and construction of the phylogenetic tree

PYL-PP2C-SnRK2 family members of *C. pepo* were identified by searching in the databases at NCBI (https://www.ncbi.nlm.nih.gov/) and CuGenDBv2 (http://cucurbitgenomics.org/v2/) by using BLASTP with the sequences of PYL-PP2C-SnRK2 on the *Arabidopsis* information resource website (https://www.arabidopsis.org/) and those provided by Boudsocq et al. [46], Xue et al. [30], and Zhao et al. [27]. The annotation of the *C. pepo* genome in CuGenDBv2 (http://cucurbitgenomics.org/v2/) was also used. SMART (http://smart.embl-heidelberg.de/) and CDD databases (https://www.ncbi.nlm.nih.gov/cdd/) were used to confirm the domains *PYL*, *PP2C* or *SnRK2* of all candidate genes identified. Candidate genes that did not contain specific domains were manually removed. The information of the identified and used *PYL*-*PP2C*-*SnRK2* genes is summarized in Tables S1-S4.

MEGA X software [85] was used to establish the phylogenetic relationships between PYL-PP2C-SnRK2 family members of *Arabidopsis* and *C. pepo*. Multiple alignments of amino acid sequences were generated using MUSCLE. Phylogenetic trees were performed using the maximum likelihood method based on the Poisson correction model, with 1000 bootstrap replicates, and the Jones–Taylor–Thornton (JTT) model.

### Analysis of gene exon-intron structures and protein-conserved motifs

The structure of the *PYL*-*PP2C*-*SnRK2* genes was predicted using CDS and genomic DNA sequences by GSDS (http://gsds.cbi.pku.edu.cn/). The conserved motifs of the PYL-PP2C-SnRK2 proteins were analyzed by MEME software (http://meme.sdsc.edu/meme/itro.html). The maximum motif number was established as 10 for the PYL and SnRK2 proteins and 20 for the PP2C proteins, and the remaining parameters were set as default values.

### Plant material and treatments

To investigate gene expression of *PYL*-*PP2C*-*SnRK2* genes in different tissues of *C. pepo*, including seed, root, corolla, ovary, meristem, leaf, and fruit, MUCU16 seeds were sown in plastic trays containing a mixture of peat and coconut fibre. After germination, the seedlings were transplanted to a greenhouse, where the seedlings grew until the plants reached full development. The tissues were collected in ice dry and stored at − 80 °C until further use. Three biological replicates were collected for each tissue, each of which was derived from 6 independent adult plants.

For the stress treatment, MUCU16 seeds were germinated in water and then transferred to plastic trays containing vermiculite. After germination, seedlings were grown for 14 days in a growth chamber with a photoperiod of 16/8 h light/dark at 24 °C and 60% relative humidity (RH) (control conditions). For cold stress, the seedlings were incubated for 48 h in a growth chamber with a photoperiod of 16/8 h of light/dark at 4 °C and 60% RH. For ABA treatment, seedling leaves were sprayed with 100 μM ABA (Sigma-Aldrich®, Cas. number: 21293–29-8, mw. = 264.32). The leaves of the ABA-treated seedling were collected 4 h after treatment. Three biological replicates were sampled for each treatment (control, cold, and ABA), each consisting of leaves from 6 independent seedlings. The collected samples were quickly frozen in dry ice and stored at − 80 °C until further use.

For the germination experiment, the MUCU16 seeds were incubated in 50 ml Falcon tubes containing 25 ml of distilled water (control) or 100 μM ABA for 16 h at 24 °C in darkness and continuous shaking. After soaking, the seeds were placed in Petri dishes covered with filter paper, moistened with 800 μL of distilled water or ABA, and incubated in a growth chamber in darkness at 24 °C and 80% HR. Samples were collected after soaking in water or 100 μM ABA for 16 h, and immediately after radicle emergence at the end of germination under each condition. Germination of each seed was considered complete when rupture of the seed coat and radicle protrusion were observed (> 1 mm). Three biological replicates for dry, soaked, and germinated seeds were sampled, each consisting of 30 seeds. The seed coat was removed before being pulverized in liquid nitrogen and stored at − 80 °C.

To select the ABA concentration, we conducted two separate experiments. First, we analyzed the percentage of germination at various concentrations of ABA in the MUCU16 inbred line. The results of the dose-response curve are illustrate in Fig. S3. We chose the ABA concentration of 100 μM because it resulted in a 35% reduction in germination compared to seeds germinated in water (Fig. S3). At higher concentrations, germination was completely blocked. Following the selection of the ABA concentration for germination, we investigated whether the application of 100 μM ABA could also impact the water loss in the leaves of MUCU16. Our findings revealed that treatment with 100 μM of ABA reduced water loss in ABA-treated seedlings at 1 and 4 h after starting treatment (Fig. S4). Additionally, cold stress also decreased the loss of water compared to the control from 1 to 24 h after the onset of treatment (Fig. S4).

Water loss assays were conducted on 14-days-old seedlings. Twelve plants per treatment were sprayed with distilled H_2_O or 100 μM ABA and placed in a growth chamber with a photoperiod of 16/8 h of light/dark at 24 °C and 60% RH. For cold treatment, the aerial parts of the seedlings were incubated at 4 °C in a growth chamber with a photoperiod of 16/8 h of light/dark and 60% RH. The weights of the aerial parts of the plants were measured at 1, 4, 6, and 24 h after the initiation of treatment. The percentage of water loss was calculated using the following formula:

$$\% Waterloss=\frac{\left({W}_{T0}-{W}_{Tx}\right)}{W_{T0}}x100$$

where W_T0_ is the initial weight (g) and W_Tx_ is the weight at each recorded point (g).

### RNA extraction and sequencing

Frozen tissue at − 80 °C was ground using stainless steel beads, previously cooled with dry ice. For RNA extraction, the E.Z.N.A® Plant RNA Kit (Omega Bio-tek) was used following the manufacturer’s protocol. After extraction, RNA was eluted in nuclease-free water and immediately prepared for sequencing on the BGI DNBseq Sequencing Platform, generating 150 pb pair-end reads. All raw reads generated were made publicly available in the NCBI database (https://www.ncbi.nlm.nih.gov/) under project number PRJNA1042934 and PRJNA1019290.

### Bioinformatic analysis of transcriptomic data

The quality of the sequenced reads was checked by the FastQC tool [86]. SOAPnuke [87] were used to delete and trim low quality bases within the data. Mapping of high-quality reads and transcriptome assembly were carried out by HISAT2 [88] and STRINGTIE [89, 90].

To evaluate the expression patterns of the *PYL*-*PP2C*-*SnRK2* genes in different tissues, gene expression levels were calculated as fragments per kilobase million (FPKM). Subsequently, a heatmap was created for each family of genes using TBtools [91]. Data were normalized using log_2_ (FPKM), and values of FPKM < 1 were considered as 1.

Differential expression analysis was performed using the total count matrix, using edgeR ver. 3.28 [92, 93] and limma-voom ver. 3.42.2 [94, 95] packages in R [96]. Voom function, available in the limma package, was applied during data treatment. The adjusted *p*-value for each gene was calculated using the Benjamini & Hochberg (BH) method [97].

Counts per million (CPM) values were calculated and used for multidimensional scaling (MDS) of the expression data using glimmaR ver. 2.10 [98]. To determine differentially expressed genes (DEG) under different treatments, only genes with adjusted P.value < 0.05 were considered.

### Supplementary Information

**Supplementary Material 1.**

**Supplementary Material 2.**

## Data Availability

All sequence information regarding *C. pepo* is available from CuGenDBv2 (http://cucurbitgenomics.org/v2/) and the accession numbers are listed in Tables S1-S3. The PYL, PP2C, and SnRK2 protein sequences from Arabidopsis are available from TAIR (https://www.arabidopsis.org/) and the accession numbers are listed in Table S4. The transcriptomic data were deposited in NCBI-SRA database (https://www.ncbi.nlm.nih.gov/sra/) (Project number: PRJNA1042934 and PRJNA1019290).
