# Supplementary material for: Structural and functional characterization of genes PYL-PP2C-SnRK2s in the ABA signalling pathway of Cucurbita pepo
Source: BMC Genomics. 2024 Mar 11;25:268. doi: 10.1186/s12864-024-10158-9 (PMC10926676; doi:10.1186/s12864-024-10158-9)
Supplement: Supplementary file 1 [file 12864_2024_10158_MOESM1_ESM.docx]

Supplementary Figures

Fig. S1

Fig. S2

Figure S2. Multidimensional scaling plot generated with glimmaR from RNA-seq data at three stages of seed germination. Each point represents 1 sample (consisting of 30 seeds). Dimensions 1 and 2 explained 55 and 17% of the variation in gene expression, respectively. Orange, dry seed; blue, seed soaked in water for 16 h (imbibition phase); pink, seed soaked in water for 16 h; dark blue, germinated seed at the stage of radicle protrusion in water; green, germinated seed at the stage of radicle protrusion in ABA.

Fig. S3

Fig. S4
